# Supplementary material for: Homeolog expression quantification methods for allopolyploids
Source: Brief Bioinform. 2018 Dec 27;21(2):395–407. doi: 10.1093/bib/bby121 (PMC7299288; doi:10.1093/bib/bby121)
Supplement: Supp_bby121 [file supp_bby121.pdf]

Supplemental Text

Tony Kuo[*,†], Masaomi Hatakeyama[‡,§,¶], Toshiaki Tameshige[‖], Kentaro K. Shimizu[‡,‖] & Jun Sese[*,†,**]

1 Supplemental Methods Hexaploid

The code and commands used in this study for hexaploid wheat *T. aestivum* are shown. Python scripts **described here and the triple_homeolog.pl script** are available at: **https://github.com/tony-kuo/eagle/tree/master/scripts**. gffread is available at: https://github.com/gpertea/gffread. STAR, LAST, Kallisto and HomeoRoq can be obtained their respective project pages.

1.1 Standard approach with STAR

```
REF=Taes_genome_2017_05
GTF=refseq
GENDIR=/project/wheat/stargenome
CPU=8

STAR --runMode genomeGenerate --genomeDir $GENDIR --genomeFastaFiles $REF.fa --sjdbGTFfile $GTF.gtf --runThreadN $CPU
for i in ‘ls *_R1.fastq.gz‘; do
    F=‘basename $i _R1.fastq.gz‘
    mkdir -p ./star/star_$F
    STAR --genomeDir $GENDIR --readFilesCommand zcat --readFilesIn $F\_R1.fastq.gz $F\_R2.fastq.gz \
            --outFileNamePrefix star_$F- --runThreadN $CPU --genomeLoad NoSharedMemory \
            --outSAMstrandField intronMotif --outFilterIntronMotifs RemoveNoncanonicalUnannotated \
            --outSJfilterCountUniqueMin 3 2 2 2 --outMultimapperOrder Random --outFilterType BySJout \
            --outStd SAM | samtools view -Shb - > ./star/$F.bam
    samtools sort -o ./star/$F.refsort.bam ./star/$F.bam
    samtools index -c ./star/$F.refsort.bam

    featureCounts -T 8 -t exon -g transcript_id -a $GTF.chrA.gtf -o ./star/$F.chrA.counts.txt ./star/$F.refsort.bam
    featureCounts -T 8 -t exon -g transcript_id -a $GTF.chrB.gtf -o ./star/$F.chrB.counts.txt ./star/$F.refsort.bam
    featureCounts -T 8 -t exon -g transcript_id -a $GTF.chrD.gtf -o ./star/$F.chrD.counts.txt ./star/$F.refsort.bam
done

python scripts/tablize.py -skip 1 -a -i 0 -c 6 ./star/*.chrA.counts.txt > star.chrA.tsv
python scripts/tablize.py -skip 1 -a -i 0 -c 6 ./star/*.chrB.counts.txt > star.chrB.tsv
python scripts/tablize.py -skip 1 -a -i 0 -c 6 ./star/*.chrD.counts.txt > star.chrD.tsv

# In terms of chrA gene id
cut -f 1 homeolog.ABD.list > homeolog.A.list
python scripts/tablize.py -a homeolog.A.list star.chrA.tsv | sort -k1 > star.chrA.homeolog.tsv
awk '{print $2"\t"$1;}' homeolog.ABD.list > homeolog.B.list
python scripts/tablize.py -a homeolog.B.list star.chrB.tsv | cut -f 2,3- | sort -k1 > star.chrB.homeolog.tsv
awk '{print $3"\t"$1;}' homeolog.ABD.list > homeolog.D.list
python scripts/tablize.py -a homeolog.D.list star.chrD.tsv | cut -f 2,3- | sort -k1 > star.chrD.homeolog.tsv
```

1.2 Standard approach with LAST

```
REF=Taes_genome_2017_05
GTF=refseq
```

[*]Artificial Intelligence Research Center, AIST, 2-3-26 Aomi, Koto-ku, 135-0064, Tokyo, Japan
[†]AIST-Tokyo Tech RWBC-OIL, 2-12-1 Okayama, Meguro-ku, 152-8550, Tokyo, Japan
[‡]Department of Evolutionary Biology and Environmental Studies, University of Zurich, Winterthurerstrasse 190, CH-8057, Zurich, Switzerland
[§]Functional Genomics Center Zurich, Winterthurerstrasse 190, CH-8057, Zurich, Switzerland
[¶]Swiss Institute of Bioinformatics, Quartier Sorge - Batiment Genopode, 1015 Lausanne, Switzerland
[‖]Kihara Institute for Biological Research, Yokohama City University, 641-12, Maioka, Totsuka-ku, 244-0813, Yokohama, Japan
[**]Corresponding Author

```
lastdb8 -W 3 -uNEAR -R01 Taes $REF.fa
samtools dict -o $REF.dict $REF.fa

for i in `ls *_R1.fastq.gz`; do
    F=`basename $i _R1.fastq.gz`
    # Explicitly add pair-end number into read id
    gzip -dc $F\_R1.fastq.gz | paste - - - - | \
       perl -ne 'chomp; @t=split(/\s+/); @u=split(/\t/); print "$t[0]_1\n$u[1]\n+\n$u[3]\n";' > $F\_R1.fastq
    gzip -dc $F\_R2.fastq.gz | paste - - - - | \
       perl -ne 'chomp; @t=split(/\s+/); @u=split(/\t/); print "$t[0]_2\n$u[1]\n+\n$u[3]\n";' > $F\_R2.fastq
    lastal8 -Q1 -D100 -P8 Taes $F\_R1.fastq $F\_R2.fastq | last-split8 -c 0 -t 0.004 -d 2 -m 1 -g Taes > ./last/$F.maf
    maf-convert -f $REF.dict sam ./last/$F.maf | samtools view -Shb - > ./last/$F.maf.bam
    samtools sort -o ./last/$F.maf.refsort.bam ./last/$F.maf.bam
    samtools index -c ./last/$F.maf.refsort.bam

    featureCounts -T 8 -Q 20 -t exon -g transcript_id -a $GTF.chrA.gtf -o ./last/$F.chrA.counts.txt ./last/$F.maf.refsort.bam
    featureCounts -T 8 -Q 20 -t exon -g transcript_id -a $GTF.chrB.gtf -o ./last/$F.chrB.counts.txt ./last/$F.maf.refsort.bam
    featureCounts -T 8 -Q 20 -t exon -g transcript_id -a $GTF.chrD.gtf -o ./last/$F.chrD.counts.txt ./last/$F.maf.refsort.bam
done

python scripts/tablize.py -skip 1 -a -i 0 -c 6 ./last/*.chrA.counts.txt > last.chrA.tsv
python scripts/tablize.py -skip 1 -a -i 0 -c 6 ./last/*.chrB.counts.txt > last.chrB.tsv
python scripts/tablize.py -skip 1 -a -i 0 -c 6 ./last/*.chrD.counts.txt > last.chrD.tsv

# In terms of chrA gene id
cut -f 1 homeolog.ABD.list > homeolog.A.list
python scripts/tablize.py -a homeolog.A.list last.chrA.tsv | sort -k1 > last.chrA.homeolog.tsv
awk '{print $2"\t"$1;}' homeolog.ABD.list $>$ homeolog.B.list
python scripts/tablize.py -a homeolog.B.list last.chrB.tsv | cut -f 2,3- | sort -k1 > last.chrB.homeolog.tsv
awk '{print $3"\t"$1;}' homeolog.ABD.list > homeolog.D.list
python scripts/tablize.py -a homeolog.D.list last.chrD.tsv | cut -f 2,3- | sort -k1 > last.chrD.homeolog.tsv
```

1.3 Kallisto

```
REF=refseq.fa
IND=Taes_kallisto

kallisto index -i $IND $REF

for i in `ls *_R1.fastq.gz`; do
    F=`basename $i _R1.fastq.gz`
    kallisto quant --pseudobam -i $IND -o kallisto_$F $F\_R1.fastq.gz $F\_R2.fastq.gz | samtools view -F 4 -Shb - > $F.bam

    grep 'TraesCS.A' kallisto_$F/abundance.tsv | cut -f 1,4 > $F.chrA.counts.txt
    grep 'TraesCS.B' kallisto_$F/abundance.tsv | cut -f 1,4 > $F.chrB.counts.txt
    grep 'TraesCS.D' kallisto_$F/abundance.tsv | cut -f 1,4 > $F.chrD.counts.txt
done
```

1.4 Homeolog identification

```
CPU=8
REF=Taes_genome_2017_05
GTF=iwgsc_refseqv1.0_HighConf_UTR_2017May05

# Extract transcript sequences
gffread -T -o refseq.gtf $GTF.gff3 # gff to gtf
gffread -g $REF.fa -w refseq.fa $GTF.gff3

grep '^chr.A' refseq.gtf > refseq.chrA.gtf
grep '^chr.B' refseq.gtf > refseq.chrB.gtf
grep '^chr.D' refseq.gtf > refseq.chrD.gtf

gffread -g $REF.fa -x chrA.cds.fa refseq.chrA.gtf
gffread -g $REF.fa -x chrB.cds.fa refseq.chrB.gtf
gffread -g $REF.fa -x chrD.cds.fa refseq.chrD.gtf

lastdb -uNEAR -R01 chrA_db chrA.cds.fa
lastdb -uNEAR -R01 chrB_db chrB.cds.fa
lastdb -uNEAR -R01 chrD_db chrD.cds.fa

lastal chrA_db -P$CPU -D10000000000 chrB.cds.fa | last-map-probs -m 0.49 > A.B.maf
lastal chrB_db -P$CPU -D10000000000 chrA.cds.fa | last-map-probs -m 0.49 > B.A.maf
```

```
lastal chrB_db -P$CPU -D10000000000 chrD.cds.fa | last-map-probs -m 0.49 > B.D.maf
lastal chrD_db -P$CPU -D10000000000 chrB.cds.fa | last-map-probs -m 0.49 > D.B.maf

lastal chrA_db -P$CPU -D10000000000 chrD.cds.fa | last-map-probs -m 0.49 > A.D.maf
lastal chrD_db -P$CPU -D10000000000 chrA.cds.fa | last-map-probs -m 0.49 > D.A.maf

python scripts/homeolog_genotypes.py -o A.vs.B -f exon -g refseq.gtf A.B.maf B.A.maf # coordinates based on A
python scripts/homeolog_genotypes.py -o B.vs.A -f exon -g refseq.gtf B.A.maf A.B.maf # coordinates based on B

python scripts/homeolog_genotypes.py -o B.vs.D -f exon -g refseq.gtf B.D.maf D.B.maf # coordinates based on B
python scripts/homeolog_genotypes.py -o D.vs.B -f exon -g refseq.gtf D.B.maf B.D.maf # coordinates based on D

python scripts/homeolog_genotypes.py -o A.vs.D -f exon -g refseq.gtf A.D.maf D.A.maf # coordinates based on A
python scripts/homeolog_genotypes.py -o D.vs.A -f exon -g refseq.gtf D.A.maf A.D.maf # coordinates based on D

# Triple copy homeologs
perl triple_homeolog.pl A.vs.B.reciprocal_best B.vs.D.reciprocal_best A.vs.D.reciprocal_best > homeolog.ABD.list

# Subgenome unique transcripts
cat A.vs.B.reciprocal_best A.vs.D.reciprocal_best | cut -f1 | sort | uniq > A.vs.all.list
cat B.vs.A.reciprocal_best B.vs.D.reciprocal_best | cut -f1 | sort | uniq > B.vs.all.list
cat D.vs.A.reciprocal_best D.vs.B.reciprocal_best | cut -f1 | sort | uniq > D.vs.all.list
python scripts/tablize.py -v0 A.vs.all.list chrA.cds.list > chrA.only.list
python scripts/tablize.py -v0 B.vs.all.list chrB.cds.list > chrB.only.list
python scripts/tablize.py -v0 D.vs.all.list chrD.cds.list > chrD.only.list
```

1.5 Subgenome alignment

```
REF=Taes_genome_2017_05
GTF=refseq
GENDIR=/project/wheat/stargenome
CPU=8

CHR="chrA chrB chrD"
for n in $CHR; do
    mkdir -p $GENDIR\_$n
    STAR --runMode genomeGenerate --genomeDir $GENDIR\_$n --genomeFastaFiles $REF.$n.fa --sjdbGTFfile $GTF.gtf --runThreadN $CPU
done

for n in $CHR; do
    for i in `ls *_R1.fastq.gz`; do
        F=`basename $i _R1.fastq.gz`
        mkdir -p ./$n/star_$F
        STAR --genomeDir $GENDIR\_$n --readFilesCommand zcat --readFilesIn $F\_R1.fastq.gz $F\_R2.fastq.gz \
            --outFileNamePrefix star_$F- --runThreadN $CPU --genomeLoad NoSharedMemory \
            --outSAMstrandField intronMotif --outFilterIntronMotifs RemoveNoncanonicalUnannotated \
            --outSJfilterCountUniqueMin 3 2 2 2 --outMultimapperOrder Random --outFilterType BySJout \
            --outStd SAM | samtools view -Shb - > ./$n/$F.bam
        samtools sort -o ./$n/$F.refsort.bam ./$n/$F.bam
        samtools index -c ./$n/$F.refsort.bam
        mv star_$F-* ./$n/star_$F
    done
done
```

1.6 EAGLE-RC

```
REF=Taes_genome_2017_05
GTF=refseq
CHR="chrA chrB chrD"

# Put the appropriate vcfs to the corresponding dir, i.e. A.vs.*.gtf.vcf in chrA
for n in $CHR; do
    cd $n
    for i in `ls *.refsort.bam`; do
        F=`basename $i .refsort.bam`
        for j in `ls *.gtf.vcf`; do
            V=`basename $j .gtf.vcf`
            eagle -t 8 -a $F.refsort.bam -r $REF.$n.fa -v $V.gtf.vcf --splice --rc 1> $F.$V.txt 2> $F.$V.readinfo.txt
            eagle-rc -a $F.refsort.bam --listonly -o $F.$V -v $F.$V.txt $F.$V.readinfo.txt > $F.$V.list
        done
    done
    cd ..
done
```

```
mkdir -p eagle
for i in `ls *_R1.fastq.gz`; do
    F=`basename $i _R1.fastq.gz`
    python scripts/ref3_consensus.py --pe -u -d -o eagle/$F.ref \
        -A chrA/$F.A.vs.B.list chrA/$F.A.vs.D.list \
        -B chrB/$F.B.vs.A.list chrB/$F.B.vs.D.list \
        -D chrD/$F.D.vs.A.list chrD/$F.D.vs.B.list
    eagle-rc --refonly --readlist -a chrA/$F.refsort.bam -o eagle/$F.chrA eagle/$F.ref.chrA.list
    eagle-rc --refonly --readlist -a chrB/$F.refsort.bam -o eagle/$F.chrB eagle/$F.ref.chrB.list
    eagle-rc --refonly --readlist -a chrD/$F.refsort.bam -o eagle/$F.chrD eagle/$F.ref.chrD.list
    featureCounts -T 8 -t exon -g transcript_id -a $GTF.chrA.gtf -o eagle/F.chrA.counts.txt eagle/F.chrA.ref.bam
    featureCounts -T 8 -t exon -g transcript_id -a $GTF.chrB.gtf -o eagle/F.chrB.counts.txt eagle/F.chrB.ref.bam
    featureCounts -T 8 -t exon -g transcript_id -a $GTF.chrD.gtf -o eagle/F.chrD.counts.txt eagle/F.chrD.ref.bam
done

# Double and triple homeolog counts
python scripts/tablize.py -skip 1 -a -i 0 -c 6 eagle/*.chrA.counts.txt > eagle.chrA.tsv
python scripts/tablize.py -skip 1 -a -i 0 -c 6 eagle/*.chrB.counts.txt > eagle.chrB.tsv
python scripts/tablize.py -skip 1 -a -i 0 -c 6 eagle/*.chrD.counts.txt > eagle.chrD.tsv

# Triple homeologs counts in terms of chrA gene id
cut -f 1 homeolog.ABD.list > homeolog.A.list
python scripts/tablize.py -a homeolog.A.list eagle.chrA.tsv | sort -k1 > eagle.chrA.homeolog.tsv
awk '{print $2"\t"$1;}' homeolog.ABD.list > homeolog.B.list
python scripts/tablize.py -a homeolog.B.list eagle.chrB.tsv | cut -f 2,3- | sort -k1 > eagle.chrB.homeolog.tsv
awk '{print $3"\t"$1;}' homeolog.ABD.list > homeolog.D.list
python scripts/tablize.py -a homeolog.D.list eagle.chrD.tsv | cut -f 2,3- | sort -k1 > eagle.chrD.homeolog.tsv

# Subgenome unique mapped reads
for i in `ls *_R1.fastq.gz`; do
    F=`basename $i _R1.fastq.gz`
    echo "" > dummy.txt
    eagle-rc --refonly --readlist -a chrA/$F.refsort.bam -u chrB/$F.refsort.bam,chrD/$F.refsort.bam -o eagle/$F.chrA.only dummy.txt
    eagle-rc --refonly --readlist -a chrB/$F.refsort.bam -u chrA/$F.refsort.bam,chrD/$F.refsort.bam -o eagle/$F.chrB.only dummy.txt
    eagle-rc --refonly --readlist -a chrD/$F.refsort.bam -u chrA/$F.refsort.bam,chrB/$F.refsort.bam -o eagle/$F.chrD.only dummy.txt
    featureCounts -T 8 -t exon -g transcript_id -a $GTF.chrA.gtf -o eagle/$F.chrA.only.counts.txt eagle/$F.chrA.only.ref.bam
    featureCounts -T 8 -t exon -g transcript_id -a $GTF.chrB.gtf -o eagle/$F.chrB.only.counts.txt eagle/$F.chrB.only.ref.bam
    featureCounts -T 8 -t exon -g transcript_id -a $GTF.chrD.gtf -o eagle/$F.chrD.only.counts.txt eagle/$F.chrD.only.ref.bam
done

python scripts/tablize.py -skip 1 -a -i 0 -c 6 eagle/*.chrA.only.counts.txt > eagle.chrA.only.tsv
python scripts/tablize.py -skip 1 -a -i 0 -c 6 eagle/*.chrB.only.counts.txt > eagle.chrB.only.tsv
python scripts/tablize.py -skip 1 -a -i 0 -c 6 eagle/*.chrD.only.counts.txt > eagle.chrD.only.tsv

# Subgenome unique mapped reads in subgenome unique genes (i.e. non-homeologs)
python scripts/tablize.py -a chrA.only.list eagle.chrA.tsv > eagle.chrA.only.unique.tsv
python scripts/tablize.py -a chrB.only.list eagle.chrB.tsv > eagle.chrB.only.unique.tsv
python scripts/tablize.py -a chrD.only.list eagle.chrD.tsv > eagle.chrD.only.unique.tsv
```

1.7 HomeoRoq

```
GTF=refseq
CHR="chrA chrB chrD"

for n in $CHR; do
    for i in `ls $n/*.refsort.bam`; do
        samtools view -H $i > homeoroq/$n\_$F.header
        samtools view $i | sort -k1 > homeoroq/$n\_$F.sam
    done
done

cd homeoroq
ln -sf ../chrA/*.refsort.bam ./
for i in `ls *.refsort.bam`; do
    F=`basename $i .refsort.bam`
    mkdir -p AvsB
    python homeoroq_140811/read_classify.py chrA_$F chrB_$F
    mv *_$F*_orig.sam *_$F*_ambi.sam *_$F*_common.sam *_$F*_unmapped.sam *_$F*_other.sam AvsB

    mkdir -p AvsD
    python homeoroq_140811/read_classify.py chrA_$F chrD_$F
    mv *_$F*_orig.sam *_$F*_ambi.sam *_$F*_common.sam *_$F*_unmapped.sam *_$F*_other.sam AvsD

    mkdir -p BvsD
    python homeoroq_140811/read_classify.py chrB_$F chrD_$F
```

```
    mv *_$F*_orig.sam *_$F*_ambi.sam *_$F*_common.sam *_$F*_unmapped.sam *_$F*_other.sam BvsD

    mkdir -p consensus
    python scripts/tablize.py -a -i 0-1 AvsB/chrA_$F\_orig.sam AvsD/chrA_$F\_orig.sam | cut -f 1-15 > consensus/chrA_$F.orig.sam
    python scripts/tablize.py -a -i 0-1 AvsB/chrB_$F\_orig.sam BvsD/chrB_$F\_orig.sam | cut -f 1-15 > consensus/chrB_$F.orig.sam
    python scripts/tablize.py -a -i 0-1 AvsD/chrD_$F\_orig.sam BvsD/chrD_$F\_orig.sam | cut -f 1-15 > consensus/chrD_$F.orig.sam

    featureCounts -T 8 -t exon -g transcript_id -a $GTF.chrA.gtf -o consensus/$F.chrA.counts.txt consensus/chrA_$F.orig.sam
    featureCounts -T 8 -t exon -g transcript_id -a $GTF.chrB.gtf -o consensus/$F.chrB.counts.txt consensus/chrB_$F.orig.sam
    featureCounts -T 8 -t exon -g transcript_id -a $GTF.chrD.gtf -o consensus/$F.chrD.counts.txt consensus/chrD_$F.orig.sam
done

# Double and triple homeolog counts
python scripts/tablize.py -skip 1 -a -i 0 -c 6 consensus/*.chrA.counts.txt > homeoroq.chrA.tsv
python scripts/tablize.py -skip 1 -a -i 0 -c 6 consensus/*.chrB.counts.txt > homeoroq.chrB.tsv
python scripts/tablize.py -skip 1 -a -i 0 -c 6 consensus/*.chrD.counts.txt > homeoroq.chrD.tsv

# Triple homeolog counts in terms of chrA gene id
cut -f 1 homeolog.ABD.list > homeolog.A.list
python scripts/tablize.py -a homeolog.A.list homeoroq.chrA.tsv | sort -k1 > homeoroq.chrA.homeolog.tsv
awk '{print $2"\t"$1;}' homeolog.ABD.list > homeolog.B.list
python scripts/tablize.py -a homeolog.B.list homeoroq.chrB.tsv | cut -f 2,3- | sort -k1 > homeoroq.chrB.homeolog.tsv
awk '{print $3"\t"$1;}' homeolog.ABD.list > homeolog.D.list
python scripts/tablize.py -a homeolog.D.list homeoroq.chrD.tsv | cut -f 2,3- | sort -k1 > homeoroq.chrD.homeolog.tsv
```

2 Supplemental Methods Tetraploid

The code and commands used in this study for tetraploid *A. kamchatica* are shown. Python scripts described here are available at: https://github.com/tony-kuo/eagle/tree/master/scripts. gffread is available at: https://github.com/gpertea/gffread. STAR, LAST, Kallisto and HomeoRoq can be obtained their respective project pages. Here we show just the subgenome-classification method commands as the other methods are identical to hexaploid described above.

2.1 Homeolog identification

```
# The lyrata gene and scaffold ids should be modified to prepend Alyr_ or some other way make ids unique
HALREF=Ahal_v2_2
HALGTF=Ahal_v2_2
LYRREF=Alyr_v2_2
LYRGTF=Alyr_v2_2_1
CPU=8

# Extract transcript sequences
gffread -T -o $HALGTF.gtf $HALGTF.gff # gff to gtf
gffread -g $HALREF.fa -w halrefseq.fa $HALGTF.gff
gffread -T -o $LYRGTF.gtf $LYRGTF.gff # gff to gtf
gffread -g $LYRREF.fa -w lyrrefseq.fa $LYRGTF.gff

lastdb -uNEAR -R01 hal_db halrefseq.fa
lastdb -uNEAR -R01 lyr_db lyrrefseq.fa

lastal hal_db -P$CPU -D10000000000 lyrrefseq.fa | last-map-probs -m 0.49 > H.L.maf
lastal lyr_db -P$CPU -D10000000000 halrefseq.fa | last-map-probs -m 0.49 > L.H.maf

python scripts/homeolog_genotypes.py -o H.vs.L -f exon -g $HALGTF.gtf H.L.maf L.H.maf # coordinates based on hal
python scripts/homeolog_genotypes.py -o L.vs.H -f exon -g $LYRGTF.gtf L.H.maf H.L.maf # coordinates based on lyr

# Subgenome unique transcripts
cat $HALGTF.gtf | perl -ne 'chomp; m/transcript_id "(.*?)";/; print "$1\n";' | sort | uniq > H.all.list
cat $LYRGTF.gtf | perl -ne 'chomp; m/transcript_id "(.*?)";/; print "$1\n";' | sort | uniq > L.all.list
python scripts/tablize.py -v0 H.vs.L.reciprocal_best H.all.list > H.only.list
python scripts/tablize.py -v0 L.vs.H.reciprocal_best L.all.list > L.only.list
```

2.2 Subgenome alignment

```
CPU=8
HALREF=Ahal_v2_2
HALGTF=Ahal_v2_2
HALGENDIR=/project/hal/stargenome
mkdir -p $HALGENDIR

LYRREF=Alyr_v2_2
LYRGTF=Alyr_v2_2_1
LYRGENDIR=/project/lyr/stargenome
mkdir -p $LYRGENDIR

STAR --runMode genomeGenerate --genomeDir $HALGENDIR --genomeFastaFiles $HALREF.fa --sjdbGTFfile $HALGTF.gtf --runThreadN $CPU
STAR --runMode genomeGenerate --genomeDir $LYRGENDIR --genomeFastaFiles $LYRREF.fa --sjdbGTFfile $LYRGTF.gtf --runThreadN $CPU

for i in `ls *_R1.fastq.gz`; do
    F=`basename $i _R1.fastq.gz`
    mkdir -p ./hal/star_$F
    STAR --genomeDir $HALGENDIR --readFilesCommand zcat --readFilesIn $F\_R1.fastq.gz $F\_R2.fastq.gz \
        --outFileNamePrefix star_$F- --runThreadN $CPU --genomeLoad NoSharedMemory \
        --outSAMstrandField intronMotif --outFilterIntronMotifs RemoveNoncanonicalUnannotated \
        --outSJfilterCountUniqueMin 3 2 2 2 --outMultimapperOrder Random \
        --outFilterType BySJout --outStd SAM | samtools view -Shb - > ./hal/$F.bam
    samtools sort -o ./hal/$F.refsort.bam ./hal/$F.bam
    samtools index -c ./hal/$F.refsort.bam
    mv star_$F-* ./hal/star_$F

    STAR --genomeDir $LYRGENDIR --readFilesCommand zcat --readFilesIn $F\_R1.fastq.gz $F\_R2.fastq.gz \
        --outFileNamePrefix star_$F- --runThreadN $CPU --genomeLoad NoSharedMemory \
        --outSAMstrandField intronMotif --outFilterIntronMotifs RemoveNoncanonicalUnannotated \
        --outSJfilterCountUniqueMin 3 2 2 2 --outMultimapperOrder Random \
        --outFilterType BySJout --outStd SAM | samtools view -Shb - > ./lyr/$F.bam
    samtools sort -o ./lyr/$F.refsort.bam ./lyr/$F.bam
    samtools index -c ./lyr/$F.refsort.bam
```

```
    mv star_$F-* ./lyr/star_$F
done
```

2.3 EAGLE-RC

```
HALREF=Ahal_v2_2
HALGTF=Ahal_v2_2
LYRREF=Alyr_v2_2
LYRGTF=Alyr_v2_2_1

cd hal
for i in `ls *.refsort.bam`; do
    F=`basename $i .refsort.bam`
    eagle -t 8 -a $F.refsort.bam -r $HALREF.fa -v ../H.vs.L.gtf.vcf --splice --rc 1> $F.H.vs.L.txt 2> $F.H.vs.L.readinfo.txt
    eagle-rc -a $F.refsort.bam --listonly -o $F.H.vs.L -v $F.H.vs.L.txt $F.H.vs.L.readinfo.txt > $F.H.vs.L.list
done
cd ..
cd lyr
for i in `ls *.refsort.bam`; do
    F=`basename $i .refsort.bam`
    eagle -t 8 -a $F.refsort.bam -r $LYRREF.fa -v ../L.vs.H.gtf.vcf --splice --rc 1> $F.L.vs.H.txt 2> $F.L.vs.H.readinfo.txt
    eagle-rc -a $F.refsort.bam --listonly -o $F.L.vs.H -v $F.L.vs.H.txt $F.L.vs.H.readinfo.txt > $F.L.vs.H.list
done
cd ..

mkdir -p eagle
for i in `ls *_R1.fastq.gz`; do
    F=`basename $i _R1.fastq.gz`
    python scripts/ref2_consensus.py --pe -u -o eagle/$F.ref \
        -A chrA/$F.H.vs.L.list \
        -B chrB/$F.L.vs.H.list
    eagle-rc --refonly --readlist -a hal/$F.refsort.bam -o eagle/$F.H eagle/$F.ref.chrA.list
    eagle-rc --refonly --readlist -a lyr/$F.refsort.bam -o eagle/$F.L eagle/$F.ref.chrB.list
    featureCounts -T 8 -t exon -g transcript_id -a $HALGTF.gtf -o eagle/F.H.counts.txt eagle/F.H.ref.bam
    featureCounts -T 8 -t exon -g transcript_id -a $LYRGTF.gtf -o eagle/F.L.counts.txt eagle/F.L.ref.bam
done

python scripts/tablize.py -skip 1 -a -i 0 -c 6 eagle/*.H.counts.txt > eagle.H.tsv
python scripts/tablize.py -skip 1 -a -i 0 -c 6 eagle/*.L.counts.txt > eagle.L.tsv

# Homeolog counts in terms of halleri gene id
python scripts/tablize.py -a H.vs.L.reciprocal_best eagle.H.tsv | cut -f 1,3- | sort -k1 > eagle.H.homeolog.tsv
python scripts/tablize.py -a L.vs.H.reciprocal_best eagle.L.tsv | cut -f 2,3- | sort -k1 > eagle.L.homeolog.tsv

# Subgenome unique mapped reads
for i in `ls *_R1.fastq.gz`; do
    F=`basename $i _R1.fastq.gz`
    echo "" > dummy.txt
    eagle-rc --refonly --readlist -a hal/$F.refsort.bam -u lyr/$F.refsort.bam -o eagle/$F.H.only dummy.txt
    eagle-rc --refonly --readlist -a lyr/$F.refsort.bam -u hal/$F.refsort.bam -o eagle/$F.L.only dummy.txt
    featureCounts -T 8 -t exon -g transcript_id -a $HALGTF.gtf -o eagle/$F.H.only.counts.txt eagle/$F.H.only.ref.bam
    featureCounts -T 8 -t exon -g transcript_id -a $LYRGTF.gtf -o eagle/$F.L.only.counts.txt eagle/$F.L.only.ref.bam
done

python scripts/tablize.py -skip 1 -a -i 0 -c 6 eagle/*.H.only.counts.txt > eagle.H.only.tsv
python scripts/tablize.py -skip 1 -a -i 0 -c 6 eagle/*.L.only.counts.txt > eagle.L.only.tsv

# Subgenome unique mapped reads in subgenome unique genes
python scripts/tablize.py -a H.only.list eagle.H.only.tsv > eagle.H.only.unique.tsv
python scripts/tablize.py -a L.only.list eagle.L.only.tsv > eagle.L.only.unique.tsv
```

2.4 HomeoRoq

```
HALREF=Ahal_v2_2
HALGTF=Ahal_v2_2
LYRREF=Alyr_v2_2
LYRGTF=Alyr_v2_2_1

mkdir -p homeoroq
for i in `ls hal/*.refsort.bam`; do
    F=`basename $i .refsort.bam`
    samtools view -H $i > homeoroq/H_$F.header
    samtools view $i | sort -k1 > homeoroq/H_$F.sam
done
```

```
for i in `ls lyr/*.refsort.bam`; do
    F=`basename $i .refsort.bam`
    samtools view -H $i > homeoroq/L_$F.header
    samtools view $i | sort -k1 > homeoroq/L_$F.sam
done

cd homeoroq
ln -sf ../chrA/*.refsort.bam ./
for i in `ls *.refsort.bam`; do
    F=`basename $i .refsort.bam`
    python homeoroq_140811/read_classify.py H_$F L_$F

    featureCounts -T 8 -t exon -g transcript_id -a $HALGTF.gtf -o $F.H.counts.txt H_$F.orig.sam
    featureCounts -T 8 -t exon -g transcript_id -a $LYRGTF.gtf -o $F.L.counts.txt L_$F.orig.sam
done

python scripts/tablize.py -skip 1 -a -i 0 -c 6 *.H.counts.txt > homeoroq.H.tsv
python scripts/tablize.py -skip 1 -a -i 0 -c 6 *.L.counts.txt > homeoroq.L.tsv

# Homeolog counts in terms of halleri gene id
python scripts/tablize.py -a H.vs.L.reciprocal_best homeoroq.H.tsv | cut -f 1,3- | sort -k1 > homeoroq.H.homeolog.tsv
python scripts/tablize.py -a L.vs.H.reciprocal_best homeoroq.L.tsv | cut -f 2,3- | sort -k1 > homeoroq.L.homeolog.tsv
```

3 Supplemental Results

Table S1: The average reduction of unique kmers in the transcriptome reference due to the presence of homeologs by percent sequence divergence.

Homeolog Divergence	Unique Kmers Lost
1%	66.47%
2%	46.01%
3%	33.00%
4%	24.23%
5%	18.02%

Table S2: Classification performance for *A. kamchatica* using ART simulated reads from annotated gene models without divergence. LAST, HomeoRoq, and EAGLE-RC have regions which cannot be classified with confidence and are thus excluded. Whereas STAR and Kallisto includes all alignments, without attempting to distinguish between ambiguous alignments.

	Correct	Misclassified
STAR	92.45%	7.55%
LAST	82.20%	0.03%
Kallisto	92.36%	7.26%
HomeoRoq	79.18%	0.88%
EAGLE-RC	79.44%	0.05%

Table S3: ***A. halleri*** read proportion RMSD between different quantification approaches for homeologs in *A. kamchatica*.

	STAR	LAST	Kallisto	HomeoRoq	EAGLE-RC
STAR	-	0.0696	0.1773	0.0880	0.1351
LAST		-	0.1787	0.1019	0.1429
Kallisto			-	0.1901	0.2103
HomeoRoq				-	0.1299
EAGLE-RC					-

Table S4: Gene AT4G25110, an example where an uncertainty in the annotation and homeolog identification will influence read counts in EAGLE-RC. Read counts for ***A. halleri*** (top, g10309.t1) versus ***A. lyrata*** (bottom, g10840.t1).

	C1	C2	C3	S1	S2	S3
STAR	27245	37380	52394	21736	16864	31873
LAST	30151	41390	57968	23851	18569	35228
Kallisto	13896	19023	26636	11018	8559	16184
HomeoRoq	23665	32761	45881	18887	14577	27497
EAGLE-RC	**0**	**0**	**0**	**0**	**0**	**0**
STAR	28	14	40	6	16	8
LAST	27	11	40	9	16	8
Kallisto	14	6	20	5	8	4
HomeoRoq	20	13	38	5	13	8
EAGLE-RC	16	6	26	4	8	4

Table S5: Gene AT5G45850, an example where a missing reference sequence in one of the subgenomes greatly affects read counting in the standard approach as compared to the HomeoRoq and EAGLE-RC. Read counts for ***A. halleri*** (top, g07628.t1) versus ***A. lyrata*** (bottom, g25113.t1).

	C1	C2	C3	S1	S2	S3
STAR	128	79	50	42	16	2
LAST	118	72	48	36	15	2
Kallisto	66	40	25	21	8	1
HomeoRoq	118	68	45	37	14	1
EAGLE-RC	118	72	48	40	14	2
STAR	**55**	**112**	**75**	**30**	**50**	**27**
LAST	**218**	**512**	**429**	**257**	**341**	**215**
Kallisto	**186**	**446**	**378**	**190**	**314**	**178**
HomeoRoq	8	26	14	2	3	0
EAGLE-RC	8	26	16	2	2	0

Table S6: Runtime comparison for hexaploid wheat, per sample. 2400 Mhz processors, 8 CPUs where applicable (HomeoRoq classification and all consensus steps are single thread). Multiple values listed per column indicate parallel processing. The total for parallel processed is thus the total of the maximum in each step.

	Alignment (hr)	Pair-wise Classification (hr)	Consensus (hr)	Total (hr)
STAR	0.32			0.32
LAST	4.00			4.00
Kallisto	0.03			0.03
HomeoRoq	0.38, 0.40, 0.37	0.55, 0.81, 0.77	1.37	2.58
EAGLE-RC	0.38, 0.40, 0.37	1.99, 1.73, 2.02, 1.86, 1.78, 1.78	0.49	2.91

Table S7: Chromosome A read proportion RMSD between different quantification approaches for homeologs in *T. aestivum*.

	STAR	LAST	Kallisto	HomeoRoq	EAGLE-RC
STAR	-	0.0802	0.2391	0.1052	0.1173
LAST		-	0.2371	0.1239	0.1306
Kallisto			-	0.2521	0.2535
HomeoRoq				-	0.1299
EAGLE-RC					-

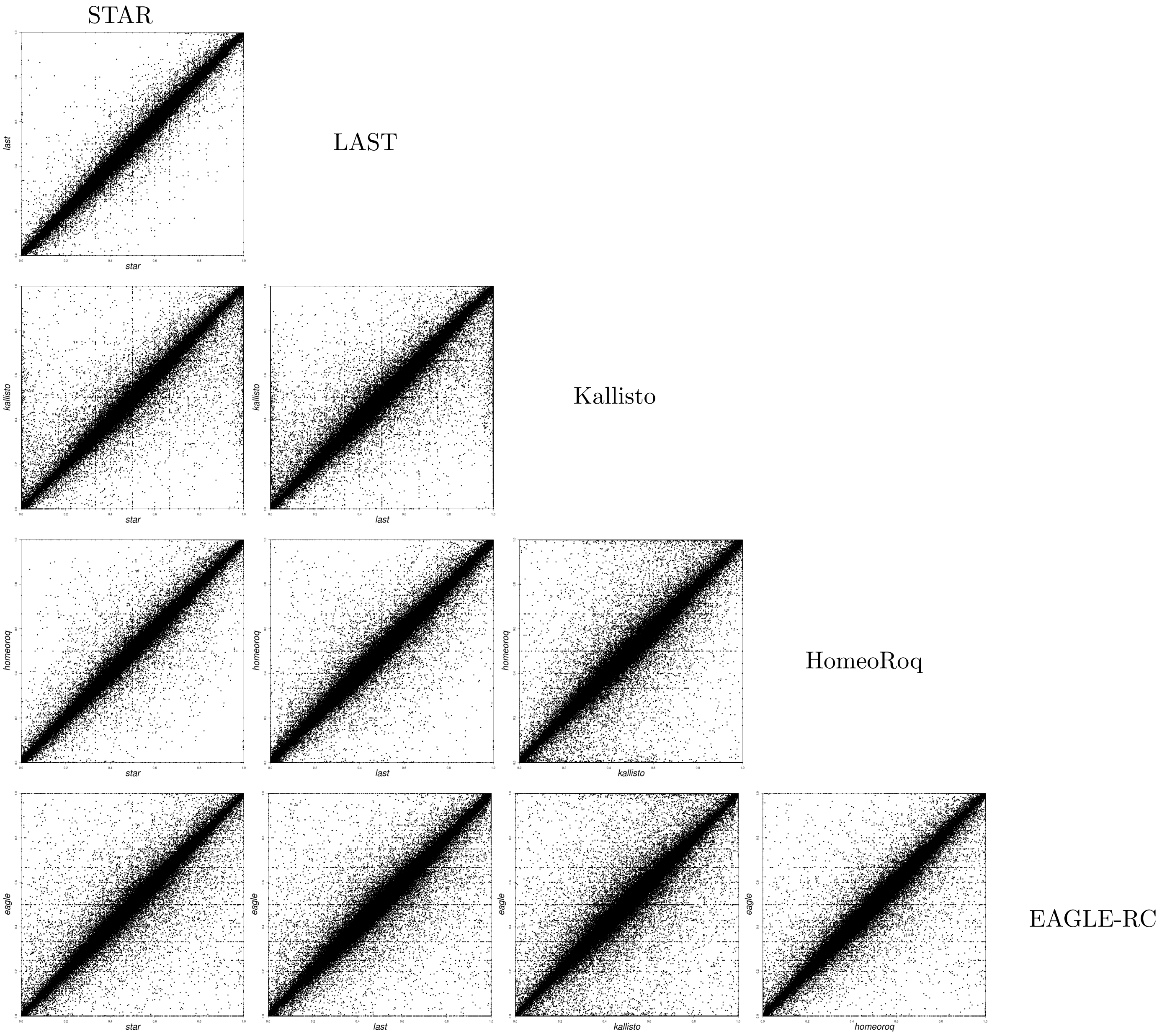

Figure S1: Homeolog expression scatter plots for tetraploid *A. kamchatica*.

a)

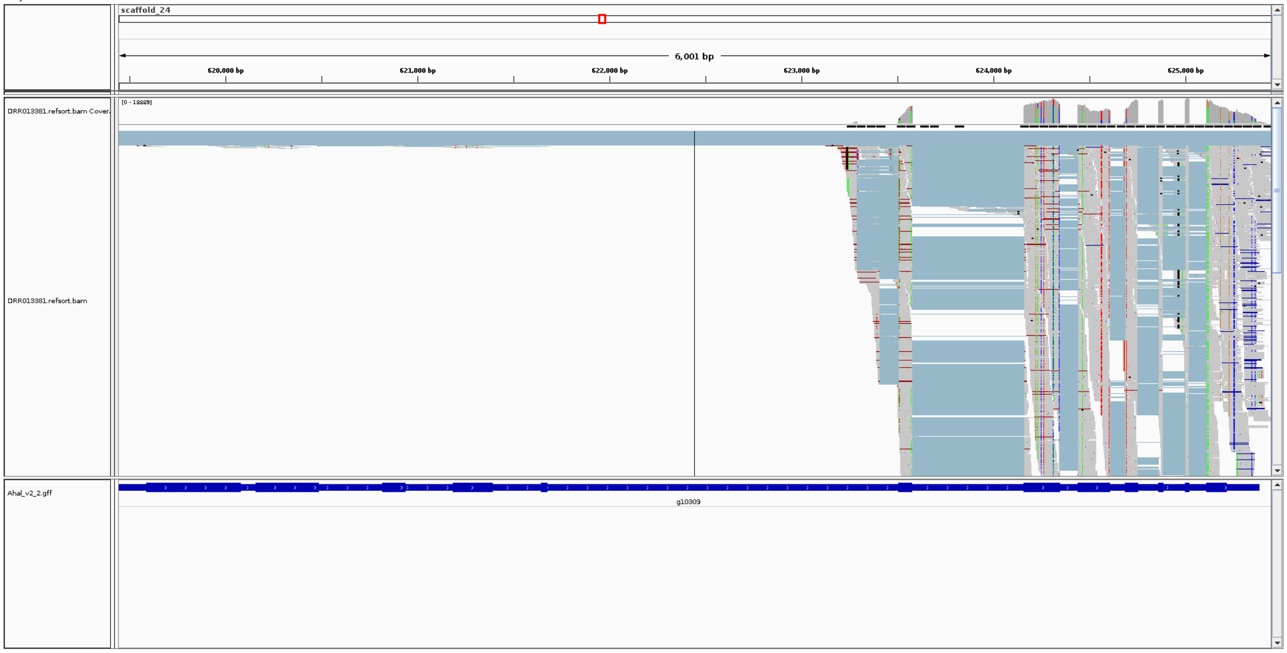

b)

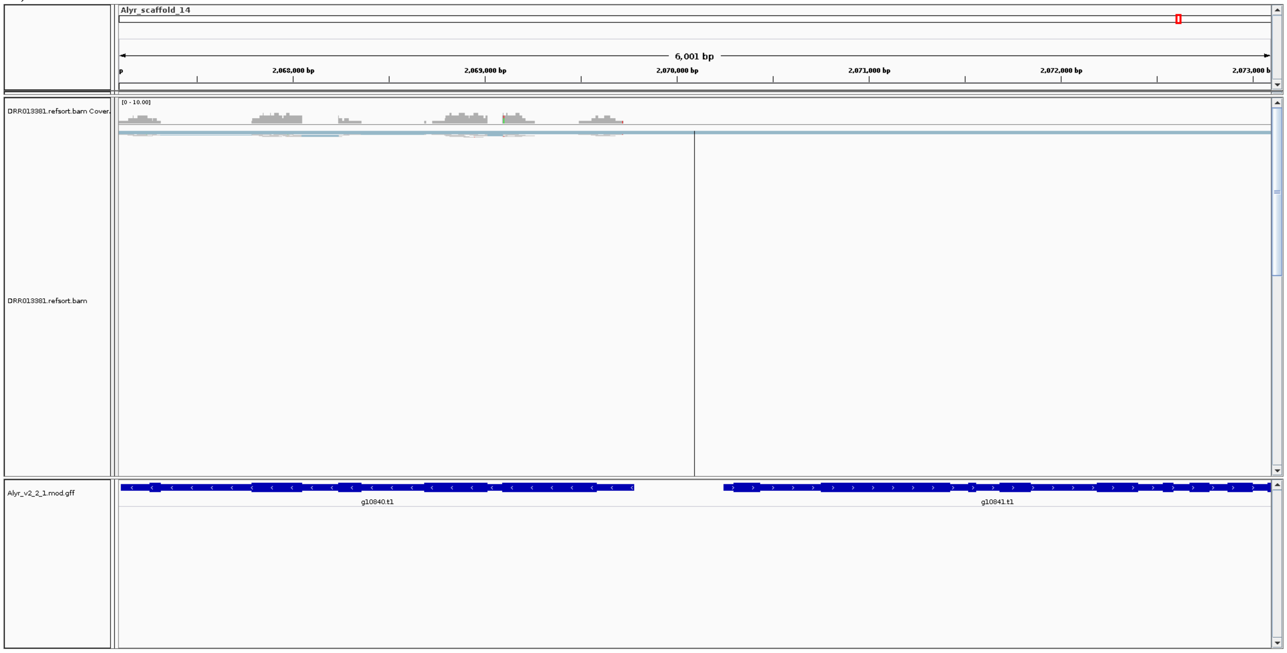

Figure S2: Annotation uncertainty for homeologs a) ***A. halleri*** g10309 and b) ***A. lyrata*** g10840. The pair-wise aligned region between these two homeologs starts from first 5' exon for 989 bp. The last 6 exons in ***A. halleri*** where the majority of reads align are uncertain in terms of whether they truly belong to this gene model.

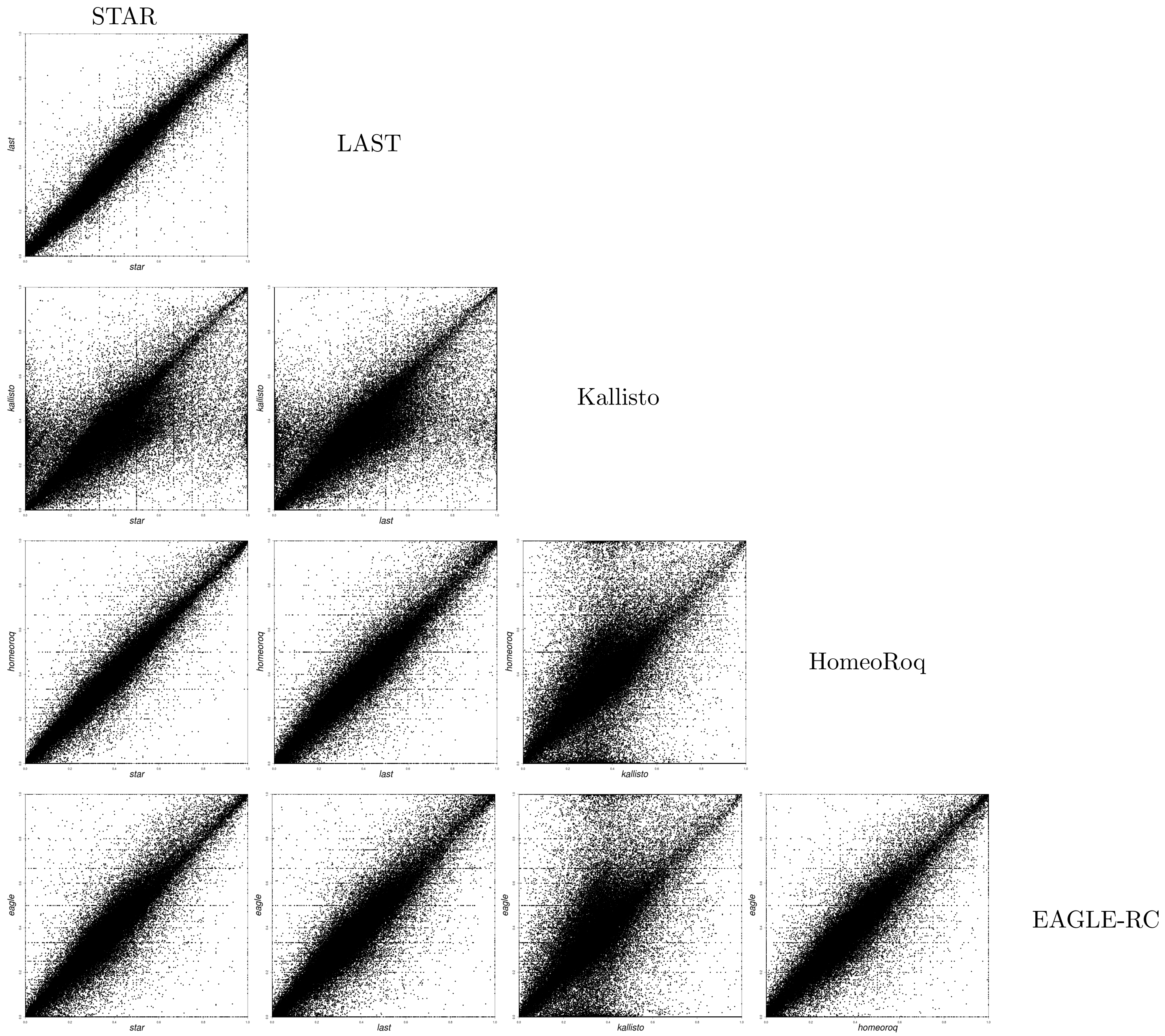

Figure S3: Homeolog expression scatter plots for hexaploid wheat, quantified as the expression proportion chromosome A over the total (A+B+D) per homeolog.

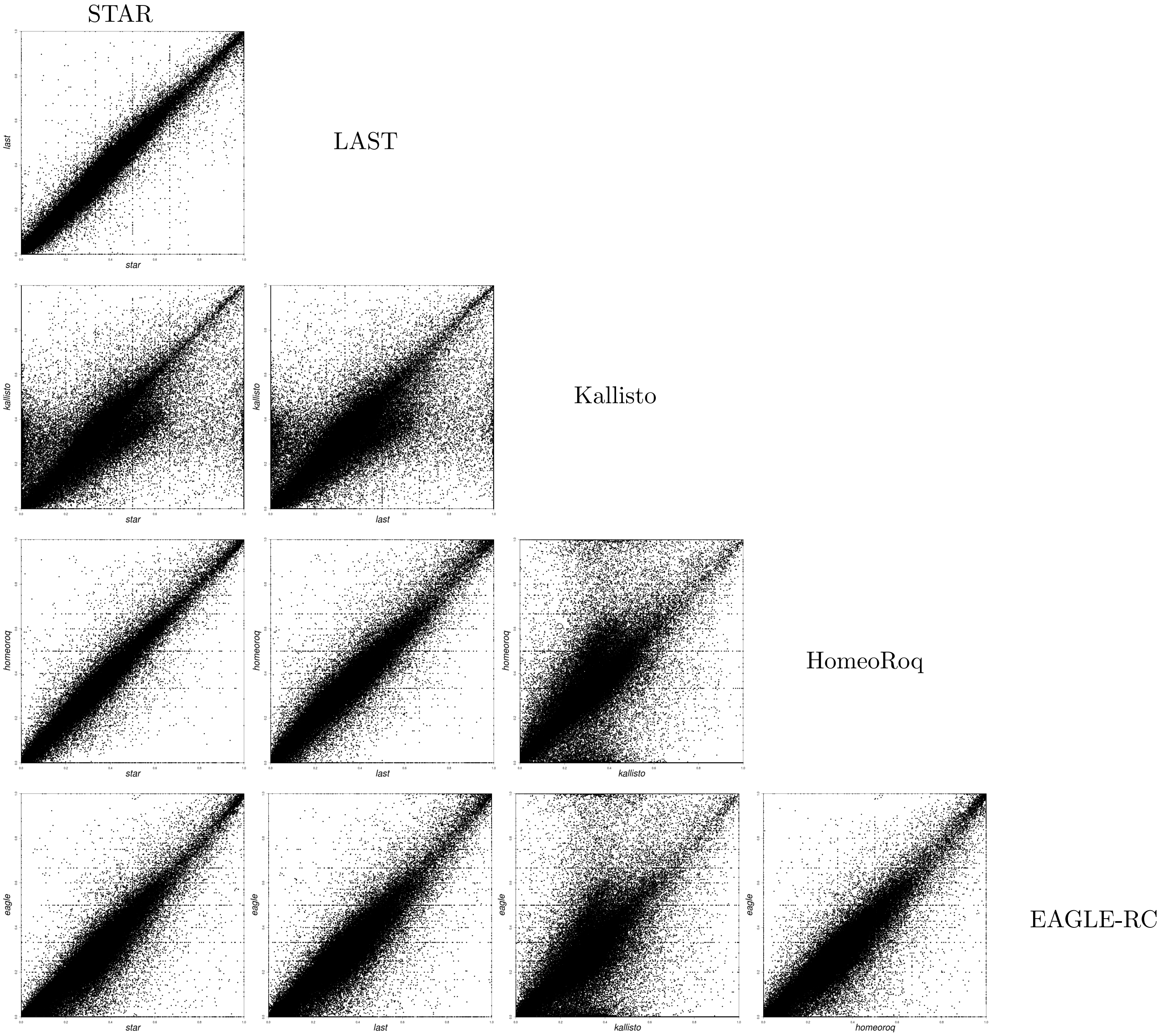

Figure S4: Homeolog expression scatter plots for hexaploid wheat, quantified as the expression proportion chromosome B over the total (A+B+D) per homeolog.

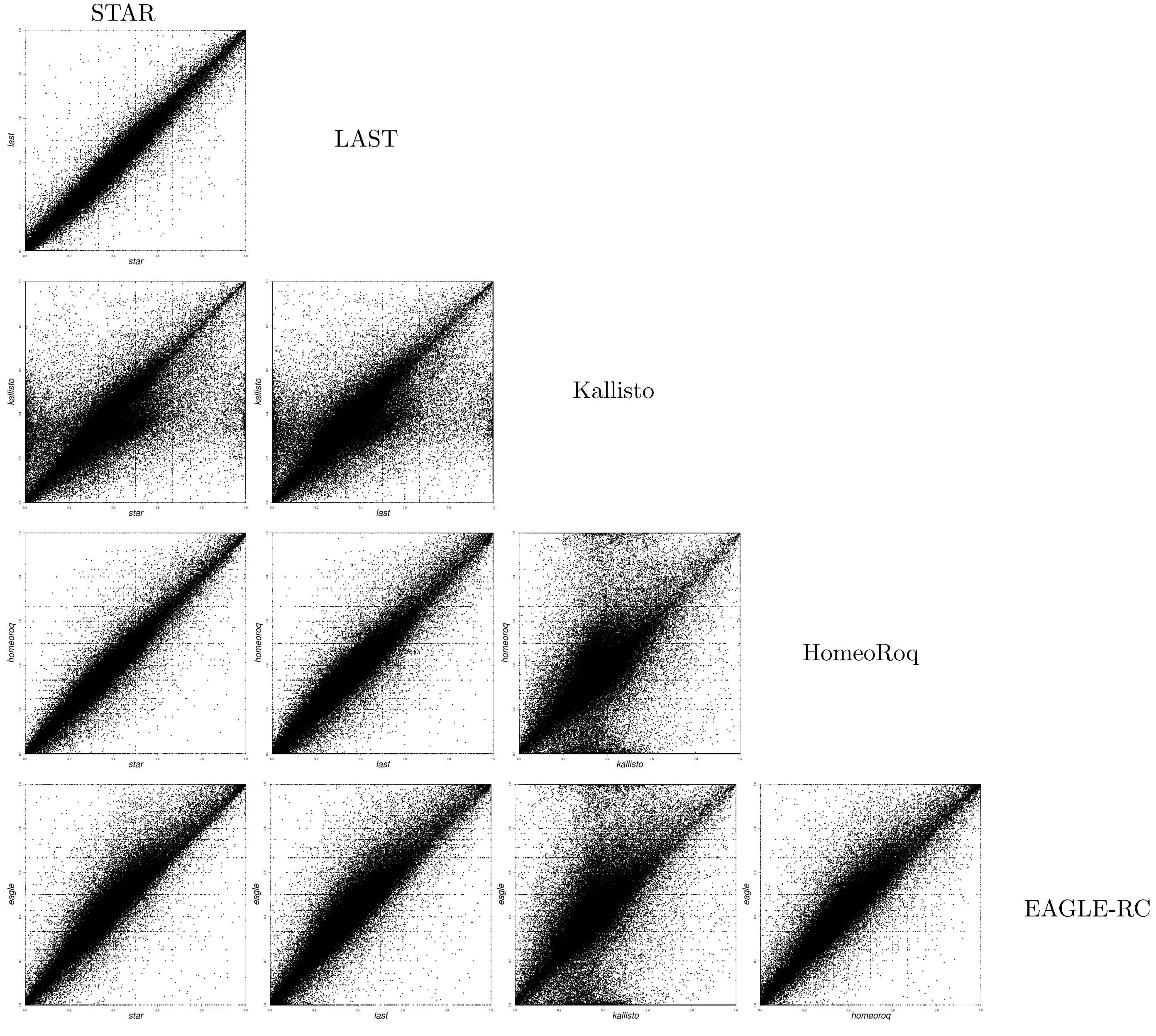

Figure S5: Homeolog expression scatter plots for hexaploid wheat, quantified as the expression proportion chromosome D over the total (A+B+D) per homeolog.
